# Lipid Metabolism and HCV Infection

**DOI:** 10.3390/v2051195

**Published:** 2010-05-11

**Authors:** Paul Targett-Adams, Steeve Boulant, Mark W. Douglas, John McLauchlan

**Affiliations:** 1 Pfizer Global Research & Development, Infectious Diseases Group, Sandwich Laboratories, Sandwich, CT13 9NJ, UK; E-Mail: Paul.Targett-Adams@pfizer.com; 2 Immune Disease Institute, Harvard Medical School, Department of Microbiology and Molecular Genetics, Boston, MA 02115, USA; E-Mail: Steeve_Boulant@hms.harvard.edu; 3 Storr Liver Unit, Westmead Millennium Institute, University of Sydney at Westmead Hospital, PO Box 412, Westmead, NSW 2145, Australia; E-Mail: mark.douglas@usyd.edu.au; 4 MRC Virology Unit, Church Street, Glasgow G11 5JR, UK

**Keywords:** hepatitis C virus, HCV, lipid metabolism, lipid droplets, fatty acid biosynthesis, cholesterol biosynthesis, VLDL assembly, RNA replication, virus assembly

## Abstract

Chronic infection by hepatitis C virus (HCV) can lead to severe liver disease and is a global healthcare problem. The liver is highly metabolically active and one of its key functions is to control the balance of lipid throughout the body. A number of pathologies have been linked to the impact of HCV infection on liver metabolism. However, there is also growing evidence that hepatic metabolic processes contribute to the HCV life cycle. This review summarizes the relationship between lipid metabolism and key stages in the production of infectious HCV.

## 1. Introduction

### 1.1. HCV epidemiology and outcomes of HCV infection

It is predicted that 2.2% of the world’s population has been infected with hepatitis C virus (HCV), an estimate that equates to approximately 130 million people [1,2]. Infection typically occurs through percutaneous exposure to infected blood. Acute infection is often symptom-free although 20 to 30% of infected persons do develop jaundice [3]. The incubation period for acute infection is thought to continue for between seven to eight weeks [4] and thereafter, up to 85% of individuals develop a chronic infection. Over a period of decades, chronic infection can lead to severe liver disease [5], including decompensated cirrhosis and hepatocellular carcinoma, which are end-stage conditions [6].

### 1.2. Molecular characteristics of HCV

HCV has been classified as a Hepacivirus within the Flaviviridae family [7,8] and separated into six genotypes or clades (numbered 1–6) [7–9]. Some genotypes have a restricted geographical distribution (genotypes 4–6) while others (genotypes 1–3) are more broadly disseminated. Although all of the genotypes can establish chronic infection, there are specific properties associated with particular genotypes. For example, steatosis is more prevalent in genotype 3 infections and there is evidence for a direct role for a viral factor in the development of this pathology [10,11]. Moreover, strains from genotypes 1 and 4 respond less well to pegylated alpha-interferon (IFN-α) and ribavirin combination therapy as compared to genotype 2 and 3 strains [12].

HCV has a positive-sense, single-stranded RNA genome of some 9.6 kilobases that encodes a polyprotein of about 3000 amino acids [13,14]. The open reading frame for the polyprotein is flanked by 5’ and 3’ untranslated regions (UTRs), which contain elements that regulate translation and replication. The polyprotein is generated by the host cell translation machinery and cleaved co- and post-translationally by viral and cellular proteases to yield the mature viral proteins. The N-terminal segment of the polyprotein encodes the structural components of the virus, which consist of core protein and two glycoproteins termed E1 and E2. Core forms the capsid shell into which the virus genome is packaged while the glycoproteins are considered to locate to the lipid envelope surrounding the capsid. However, it should be emphasized that the structure and composition of HCV virions is poorly defined since virus isolated from infectious serum is associated with lipoprotein [15,16]. A small protein called p7, which resides immediately downstream from E2, is required for virus assembly [17,18] although it is not known whether it is also a component of virions.

The C-terminal component of the polyprotein contains non-structural proteins (NS2, NS3, NS4A, NS4B, NS5A and NS5B) that provide functions necessary for synthesis of viral RNA [19,20]; several of these proteins also participate in assembly of infectious particles [17,21–25]. Expression of a polyprotein encoding NS3-NS5B, along with the 5’ and 3’ UTR elements (referred to as sub-genomic replicons), is sufficient to initiate and sustain constitutive HCV RNA replication. Viral RNA replication occurs at rearranged internal membrane structures known as the membranous web [26]. Viewed under the electron microscope, the membranous web is observed as networks of membrane-bound vesicles, derived from the ER, and similar in appearance to those observed in poliovirus-infected cells [27,28].

## 2. Cell organelles and metabolic pathways that contribute to HCV infection

Development of tissue culture systems that support replication of viral RNA and production of infectious virions has enabled identification of the cellular processes contributing to these latter phases of the HCV life cycle. Accumulating evidence has established critical roles for pathways involving lipid biosynthesis and very low density lipoprotein (VLDL) assembly as well as storage organelles, called lipid droplets. The following sections outline some of the key characteristics of the triacylglycerol and cholesterol ester biosynthesis pathways, stages in VLDL assembly and features of lipid droplets to aid understanding of their contribution to the virus life cycle.

### 2.1. Fatty acid and cholesterol biosynthesis

**Figure 1.**
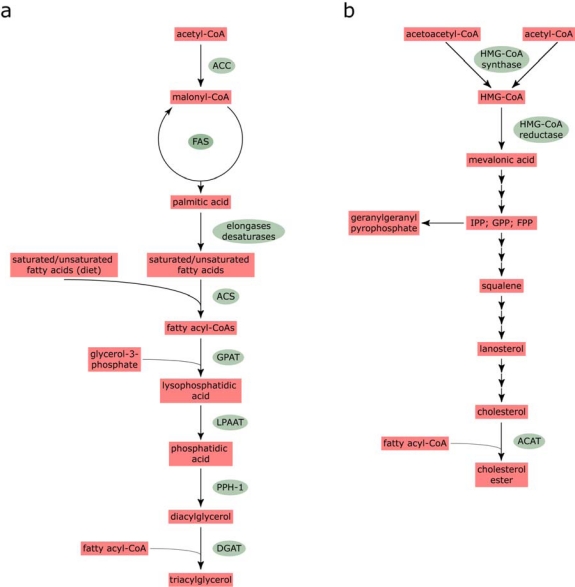
Scheme for triacylglycerol **(a)** and cholesterol ester synthesis **(b)**. For the synthesis of triacylglycerols, note that there is an alternative monoacylglycerol pathway for TAG synthesis in addition to the glycerol phosphate pathway shown in **a** [33]. In addition, fatty acids from diet are utilized for production of fatty acyl-CoAs as well as endogenously synthesized fatty acids. Abbreviations are as follows: ACAT, acyl-coenzyme A:cholesterol transferase; ACC, acetyl-CoA carboxylase; ACS, long-chain acyl-CoA synthetase; DGAT, diacylglycerol acyltransferase; FAS, fatty acid synthase; FPP, farnesyl pyrophosphate; GPAT, glycerol-3-phosphate acyltransferase; GPP, geranyl pyrophosphate; HMG-CoA, 3-hydroxy-3-methylglutaryl-CoA; IPP, isopentenyl pyrophosphate; LPAAT, lysophosphatidic acid acyltransferase; PPH-1, phosphatidate phosphohydrolase-1.

Biosynthesis of fatty acids and cholesterol is of fundamental importance to maintenance of cellular homeostasis. Fatty acid synthesis initiates through the irreversible carboxylation of acetyl-CoA by acetyl-CoA carboxylase to generate malonyl-CoA (Figure 1A). Thereafter, fatty acid synthase performs a cycle of elongation steps, utilizing one acetyl-CoA and seven malonyl-CoA moieties, to generate palmitic acid [29]. Fatty acid synthase is a large, homodimer of 552 kDa that catalyses each of the sequential reactions [30]. Palmitic acid is then employed as a substrate for further elongation or desaturation. Elongation is mediated by a family of elongase enzymes, referred to as ELOVLS (Elongation of very-long-chain fatty acids) [31] while the desaturases are responsible for introducing double bonds at specific positions in the acyl chain of fatty acids. Mammalian cells express three desaturases (Δ9, Δ6 and Δ5); the Δ9-desaturase is commonly referred to as stearoyl-CoA desaturase while the Δ6- and Δ5-desaturases are termed fatty acid desaturases. Elongases exhibit preferential specificity for saturated, mono- and poly-unsaturated fatty acids and hence there is complex interplay between the ELOVLS and desaturases.

Incorporation of fatty acids into triacylglycerols (TAGs), which are major components of the hydrophobic core in lipid droplets (see below), begins with the addition of an acyl-CoA ester by long-chain acyl-CoA synthetase (ACS; Figure 1A). Thereafter, two fatty acyl-CoAs are used to sequentially acylate glycerol-3-phosphate, firstly forming lysophosphatidic acid (catalyzed by glycerol-3-phosphate acyltransferase) and then phosphatidic acid (catalyzed by lysophosphatidic acid acyltransferase). Phosphatidic acid is converted to diacylglycerol by phosphatidate phosphohydrolase-1 before final addition of another fatty acyl-CoA to yield TAG. This final stage in the pathway is performed by diacylglycerol acyltransferase (DGAT). Two DGAT enzymes, DGAT1 and DGAT2, have been identified, which appear to have distinct intracellular roles [32]. It has been hypothesized that DGAT1 may be involved in esterification of exogenous fatty acids or the re-esterification of fatty acids after their hydrolysis. By contrast, DGAT2 may incorporate endogenously synthesized fatty acids into triglycerides.

Cholesterol biosynthesis is more complex than the pathway for production of fatty acids and involves at least 30 distinct enzymatic reactions. Synthesis can be divided into five major steps (Figure 1B). Firstly, HMG-CoA synthase catalyzes a condensation reaction between acetyl-CoA and acetoacetyl-CoA to yield 3-hydroxy-3-methylglutaryl-CoA (HMG-CoA). The second stage is the production of mevalonic acid from HMG-CoA by HMG-CoA reductase, the rate-limiting step in the cholesterol synthesis pathway and the target for statins. Following a combination of phosphorylation and decarboxylation reactions, mevalonate gives rise to isopentenyl pyrophosphate (IPP), which is then converted to squalene by a series of condensation and reduction reactions. The pathway between IPP and squalene includes the production of geranyl pyrophosphate (GPP) and farnesyl pyrophosphate (FPP); these intermediates are used not only in the squalene synthesis pathway but also for the production of geranylgeranyl pyrophosphate (GGPP), which is a substrate for geranylgeranylation of proteins [34]. Conversion of squalene to cholesterol occurs through a complex pathway that includes lanosterol as a major intermediate. Subsequent formation of cholesterol esters (CE; Figure 1B) from cholesterol and fatty acyl-CoAs is mediated by acyl-coenzyme A:cholesterol transferase (ACAT). Two ACAT genes have been identified, ACAT1 and ACAT2, both of which are expressed in hepatocytes [35]. Along with TAG, CE is stored in the hydrophobic core of lipid droplets (see below).

### 2.2. Characteristics of lipid droplets

Lipid droplets are cytoplasmic storage organelles that are composed principally of a hydrophobic core of TAG and CE. Bounding the hydrophobic core is a monolayer of phospholipid whose composition is distinct from that of the rough endoplasmic reticulum (ER) and cholesterol/sphingolipid microdomains [36]. The phospholipid layer is coated in a surface layer of proteins, which have a wide array of functions including roles in lipid metabolism, intracellular trafficking and signaling [37]. Lipid droplets are not a homogeneous population of organelles and can differ in protein composition between different cell types and by altering the metabolic state of cells [38]. Indeed, within individual cells, it is likely that distinct populations of lipid droplets exist, raising the possibility that there are pools of lipid droplets with differing functions.

### 2.3. Assembly of VLDL

VLDLs are released by hepatocytes in the liver and have a similar architecture to lipid droplets in that they are composed of a TAG- and CE-rich hydrophobic core that is bounded by a layer of phospholipid and protein [39]. The proteins that coat VLDL consist of non-exchangeable (apolipoprotein B [apoB]) and exchangeable apoproteins (principally apoCI-III and apoE) [40]. ApoB is a long, amphipathic protein of 4536 amino acids [41,42] that is essential for assembly of VLDL. Assembly is considered to proceed through two stages of lipidation of apoB. Firstly, apoB is lipidated by microsomal transfer protein (MTP), which is an ER lumenal protein. MTP is a heterodimer of two proteins with molecular weights of 88 and 58 kDa, the smaller of which is protein disulfide isomerase [43]. MTP both binds to apoB and facilitates transfer of lipid to the protein during its translation, ultimately leading to the production of a precursor VLDL (pre-VLDL) particle that is poorly lipidated [44]. Bulk addition of lipid constitutes the second stage in VLDL assembly, generating a mature, secretion-competent particle. However, the intracellular location and processes involved in lipidation of pre-VLDL are not well understood. It appears that the further addition of lipid to pre-VLDL does not rely on either MTP or the need for ongoing TAG synthesis [45,46]. Indeed, all the available evidence indicates that lipid droplets supply the lipid that is needed to convert pre-VLDL to a mature particle [47]. By way of example, increasing the expression levels of adipocyte differentiation-related protein (ADRP), the major protein on the surface of lipid droplets in hepatocyte-derived cells [48], stimulates accumulation of droplets and reduces secretion of VLDL from rat hepatocytes [49].

Mobilization of lipid from droplets relies on several enzymes involved in TAG and cholesterol metabolism. Transfer of TAG and CE from a lipid droplet to pre-VLDL seems to require de-esterification followed by re-esterification. A number of reports have investigated involvement of several enzymes including triacylglycerol hydrolase (TGH), DGAT1, and ACAT2. TGH converts TAG to acylglycerols and fatty acids [50]. Expression of TGH in mice and rat hepatoma cells gives a modest rise in triglyceride secretion [50,51]. As stated above, DGAT1 catalyzes the last stage in the synthesis of TAG and there is evidence to suggest that the enzyme participates in hepatic VLDL assembly [52]. By contrast, DGAT2 appears to direct TAG for lipid storage [52]. ACAT2 plays a major contribution to CE formation in the liver and intestine [53,54]. Studies in rat cells have shown that overexpression of human DGAT1 and ACAT2 individually leads to increased secretion of VLDL [55]. Interestingly, compared to ACAT1, ACAT2 induced higher levels of VLDL secretion, thereby providing evidence for its predominant role in CE formation in the liver. However, it should be noted that there are conflicting data from overexpression studies in rodents, [for example [56]], which illustrates the difficulty with formal demonstration that any individual enzyme can stimulate VLDL secretion.

## 3. HCV proteins that attach to lipid droplets

Before the availability of a tissue culture system that produced infectious virus particles, two viral proteins, core and NS5A, had been reported to attach to lipid droplets. Recent studies have established that these lipid droplet-binding characteristics are vital for virus assembly. Hence, the following sections describe some of the essential properties of both proteins, particularly in relation to their ability to attach to lipid droplets.

### 3.1. Properties of the HCV core protein

As stated in Section 1.2, core protein forms the capsid within HCV virions. Maturation of core requires cleavage by two cellular proteases at a signal peptide between core and the adjacent E1 glycoprotein [57–59]. The first proteolytic event is performed by signal peptidase, which cuts between amino acids 191 and 192 in the ER lumen, thereby generating an immature form of core and the N-terminus of E1. The fully processed form of core requires further proteolysis by signal peptide peptidase within the signal peptide sequence at the C-terminus of immature core. The precise location of signal peptide peptidase cleavage remains unclear, but analysis of core protein isolated from cells indicates that its C-terminus lies between amino acids 177 and 182 [57,60].

Mature core can be separated into two domains, termed D1 and D2 [61]. D1 consists of the N-terminal 117 amino acids of core and contains a high proportion of positively-charged residues. D1 is thought to interact with viral RNA but also associates with a variety of cellular components [62]. D2 begins at amino acid position 118 and terminates at the C-terminus of the protein. This domain is responsible for directing core to lipid droplets and does not require sequences in D1 for attachment. Indeed, D1 can be replaced by a heterologous protein with no disruption to lipid droplet-targeting by D2 [63]. D2 and its membrane-binding characteristics also contribute to folding of the entire core protein [64]. Structural studies on the domain have revealed that it contains two amphipathic α-helices (Helix I and Helix II) that are separated by a non-structured segment with several hydrophobic residues, termed the hydrophobic loop (HL) [63]. Removal of segments from D2 that includes deletion of these structural features leads to loss of lipid droplet localization of core. Moreover, mutations at individual amino acids on the hydrophobic faces of HI and HII can abolish attachment to droplets. Such substitutions can also induce degradation of core by the proteasome [63]. Thus, it has been proposed that attachment of D2 to membranous surfaces is critical for folding of the domain, which in turn enables folding of the remainder of core, and disruption of membrane binding exposes core to cellular degradative processes.

### 3.2. Properties of the HCV NS5A protein

NS5A is a phosphoprotein that is detected in basally phosphorylated (56 kDa) and hyperphosphorylated (58 kDa) forms. It is composed of three domains (DI-DIII) that are separated by low complexity sequences LCSI (between DI and DII) and LCSII (between DII and DIII), which are trypsin-sensitive [65]. Domains DI and DII are essential for HCV RNA replication while recent studies have demonstrated that DIII plays a key role in virion assembly [21,24,66–68]. The crystal structure of DI suggests that NS5A forms a dimer that contains a groove for binding RNA at the interface between the monomers [69]. The N-terminal end of DI also has an amphipathic helix that anchors the protein to membranes [70,71]. In contrast to DI, there is no structural information available for either DII or DIII.

Compared with core protein, NS5A is less readily detected on lipid droplets and is more broadly distributed on the ER membrane [72]. There is limited information regarding the motifs that are needed for attachment of NS5A to lipid droplets. However, it has been noted that mutation of residues between amino acids 99–104 in DI impairs detection of NS5A on droplets [73]. These residues are located in a segment of random coil at the junction of two subdomains in DI, which is relatively proline-rich. Moreover, mutations in DIII that do not disrupt lipid droplet targeting reduce the extent of co-localization between NS5A and core on droplets [21]. Although these data indicate that NS5A does not require core for lipid droplet attachment, additional studies are required to identify in greater detail the droplet-targeting elements in the protein.

## 4. The contribution of fatty acid and cholesterol biosynthesis to HCV RNA replication

Microarray studies in chimpanzees have indicated that both acute and chronic HCV infection correlates with changes in the expression patterns of genes, which have either a direct role in lipid metabolism or regulate the fatty acid and cholesterol biosynthesis pathways [74–76] suggesting a link between viremia and lipid metabolism. Direct evidence from tissue culture models that lipid biosynthesis contributes to replication of the HCV genome emerged from analysis of changes in viral RNA mediated by modulators and inhibitors of fatty acid and cholesterol metabolism [76–78]. In the cholesterol biosynthesis pathway, there has been particular focus on statins, since these compounds are potent inhibitors of HMG-CoA reductase and are widely used as a therapeutic to lower blood cholesterol levels. Early studies established that lovastatin reduced HCV RNA levels by more than 20-fold in cells harboring viral replicons [77,78]. Subsequent comparative analysis of a range of statins demonstrated that mevastatin, simvastatin and atorvastatin were more potent inhibitors of HCV RNA replication than lovastatin [79,80]; fluvastatin had either equivalent [79] or greater potency [80] compared to lovastatin. Interestingly, in neither of these studies did pravastatin have any effect on viral RNA levels, suggesting that inhibition of HCV RNA synthesis may not result solely from effects of statins on HMG-CoA reductase [80]. Combining statins with either IFN-α or anti-HCV compounds enhances reduction in HCV RNA levels and can delay the emergence of resistance mutants in tissue culture cells [79,80]. These data provide promise that statins could be combined with the current standard treatment regimens to improve response to therapy. However, early clinical trials have given equivocal results. Monotherapy with fluvastatin has been reported to give moderate and short-lived reductions in viral load [81] while statins apparently improve ALT levels [82] and, in combination with IFN-α and ribavirin, enhance the rapid virological response [83]. However, other studies report no improvement in sustained clearance of virus following use of statins as an adjunct to current treatment regimens [83–85].

Interestingly, the inhibitory effect of statins on RNA replication in tissue culture cells can be alleviated by addition of geranylgeraniol [78] and inducing the accumulation of geranylgeranyl intermediates gives higher levels of viral RNA synthesis [77]. These results led to identification of FBL2, which is modified by geranylgeranylation (see Section 2.1), as a cellular factor that binds to HCV NS5A, thereby promoting HCV RNA synthesis through a mechanism that is, as yet, unknown [86].

Compared to components engaged in cholesterol metabolism, the role of the fatty acid biosynthesis pathway in HCV RNA replication has received somewhat less attention. Inhibition of acetyl-CoA carboxylase, which irreversibly converts acetyl CoA to malonyl CoA, gives a three-fold decrease in viral RNA replication [77]. Treatment of cells harboring the HCV replicon with cerulenin, a fatty acid synthase inhibitor, also yields a modest lowering of HCV RNA levels [76]. In agreement with a contribution of fatty acid biosynthesis to RNA replication, the abundance of viral RNA increased upon addition of saturated and monounsaturated fatty acids to cells [77]. By contrast, polyunsaturated fatty acids (PUFAs; e.g., arachidonic, eicosapentaenoic and docosahexaenoic acids) lowered HCV genome synthesis [77,87]. PUFAs can suppress lipogenesis in the liver however impairment of HCV RNA replication does not apparently operate by this mechanism [77] but rather through production of lipid peroxides from exogenously administered PUFAs [88].

## 5. The contribution of lipid droplets to production of infectious HCV

### 5.1. Lipid droplets are associated with HCV-induced membrane rearrangements

It is widely accepted that the membranous web houses sites of HCV genome synthesis (see Section 1.2). In a recent study, a higher percentage of lipid droplets was observed in close proximity to modified ER membrane cisternae in Huh-7 cells that contained replicating full-length JFH-1 genomes compared to naïve cells [73]. Specifically, rearranged ER membranes appeared to partially enclose lipid droplets in a configuration more reminiscent of the convoluted membrane structures observed in cells infected with Kunjin virus (a member of the *Flaviviridae* family) [73,89]. Core protein was observed mainly on the periphery of lipid droplets and although some NS5A protein was detected on the lipid droplet surface, the majority was distributed at sites distal to droplets [73]. Furthermore, 50 nm virus-like particles, which reacted with core- and E2-specific antibodies, were also observed in close proximity to lipid droplet-associated membranes, suggesting infectious HCV particles were generated from such a membranous environment [73]. In a separate study, the interaction of HCV-like particles with lipid droplets was assessed using three-dimensional reconstructions of serial ultrathin electron microscopy sections produced from cells producing HCV core protein [90]. The results also supported the notion that budding of virus is initiated from membranes closely associated with lipid droplets [90].

### 5.2. Virus production is dependent upon recruitment of replication complexes to lipid droplet-associated membranes

The precise purpose for attachment of core to lipid droplets remains unknown and is currently an active area of study. Historically, the absence of tissue culture systems to propagate HCV meant that the mechanisms by which core-coated lipid droplets interacted with ER-resident replication complexes to facilitate virion assembly were not amenable to investigation. However, following the discovery that HCV strain JFH-1 genotype and chimeras derived from this strain could release infectious particles from cells [91–93], it has been established that the NS proteins localize to distinct foci juxtaposed to lipid droplets in cells producing progeny virus [21,73,94–96]. These specific lipid droplet-associated foci likely represent accumulations of replication complexes since negative-sense HCV RNA and virus-specific dsRNA replicative intermediates are detected within the foci [73,94]. Replication complexes do not localize to lipid droplet-associated regions of the ER in cells containing subgenomic HCV replicons, therefore, lipid droplets are presumably not required for HCV RNA replication per se [94]. Blocking attachment of core to lipid droplets in cells containing JFH-1 genomes, through mutations in either the D2 domain or the core-E1 signal sequence to impair signal peptide peptidase cleavage, prevents detection of HCV-induced dsRNA-containing foci and NS proteins in close proximity to lipid droplets [73,97]. Under these circumstances, HCV genome replication appeared unaffected but release of infectious virus was impaired [97]. Thus, recruitment of replication complexes to lipid droplet-associated regions of the ER membrane is a phenomenon likely required for the assembly of infectious virus progeny.

There are at least two possible mechanisms in operation in HCV-infected cells, which serve to localize replication complexes to regions of the ER in close proximity to core-coated lipid droplets. The first centers on the capacity of core to induce lipid droplet redistribution [98,99]. In virus-infected cells, or cells expressing core protein alone, lipid droplets are redistributed from a diffuse cytoplasmic localization to the perinuclear region [98,99]. Lipid droplet redistribution coincides with release of infectious virus progeny in cells containing full-length JFH-1 genomes and redistribution is believed to be dependent upon the microtubule network [98]. Furthermore, in nocodazole-treated cells in which lipid droplet redistribution has been inhibited, virus release is impaired [98]. Aggregation of lipid droplets at the perinuclear region increases the level of colocalization observed between core-coated lipid droplets and ER-resident replication complexes and may effectively serve to concentrate core at sites of replication to increase the likelihood of virus assembly [94,98]. Another mechanism that could facilitate the congregation of core-coated lipid droplets and replication complexes involves the HCV-encoded NS5A protein. NS5A is a component of the HCV replication complex and is essential for viral genome replication but its precise function in the HCV life cycle remains unknown [68–71, 100–102]. However, several lines of evidence exist to support a role for NS5A in the recruitment of replication complexes to core-coated lipid droplets. Firstly, NS5A exhibits an inherent capacity to localize to the surface of droplets [72]. Furthermore, variants of NS5A containing mutations in domain DI have impaired capacity for lipid droplet-association, and also prevent recruitment of other NS proteins and viral RNA to lipid droplets leading to diminished virus production [73]. Deletions in domain DIII have also been shown to disrupt the colocalization of NS5A with core-coated lipid droplets resulting in the abrogation of infectious particle release [21]. Additionally, DIII facilitates an interaction between NS5A and core, suggesting a mechanism whereby NS5A can target replication complexes to lipid droplets via a direct interaction with core protein on the droplet surface [103]. One other option is that cellular cyclophilins, which share peptidyl-prolyl cis-trans-isomerases activity and play an important role in HCV RNA replication [104–106], may influence the mechanism for directing replication complexes to core-coated lipid droplets although this possibility has not been tested. Taken together, the combined actions of core and NS5A in virus-infected cells likely play pivotal roles in the accumulation of replication complexes at lipid droplet-associated regions of the ER membrane.

### 5.3. Rationale for targeting replication complexes to lipid droplet-associated regions of the ER membrane

The requirement for replicated genomes to be available at sites of virus assembly to initiate virion morphogenesis may serve to explain the close proximity of replication complexes with lipid droplets coated with core protein [73,97]. The mechanism for transfer of replicated genomes from replication complexes to core on the surface of lipid droplets is unknown, but NS5A has several specific biological properties that appear particularly suited to such a role. Firstly, NS5A binds to the 3’ positive-sense polypyrimidine tract of HCV genomes with high affinity, a function believed to be mediated following the dimerization of the protein, which creates a putative RNA-binding cleft in the DI domains of the dimer [69,102,107]. Secondly, replicons encoding defects in NS5A can be rescued by trans-complementation with wt replicons suggesting NS5A is able to transfer between individual replication complexes [21,108,109]. This finding indicates NS5A may be a more flexible, rather than fixed, component of the replication complex, a property suited to a role in the transfer of replicated genomes from replication complexes to core at the surface of lipid droplets. Thirdly, NS5A has long been known to exist as differentially phosphorylated species in cells replicating HCV RNA [110–112], and phosphorylation of NS5A is believed to act as a regulator of events in the HCV life cycle [24,66,113]. Indeed, inhibition of NS5A hyperphosphorylation elevates levels of HCV genome replication [66,100,114,115]. Recently, it was established that phosphorylation of a serine residue located in a casein kinase II consensus motif in the DIII domain of JFH-1 NS5A was required for virion production [24]. Abrogating phosphorylation at this site perturbed production of infectious virus particles without affecting RNA replication [24]. These findings indicate that hyperphosphorylation of NS5A favors the latter stages of the HCV life cycle, perhaps by stimulating genome transfer from replication complexes to core-coated lipid droplets by promoting replication complex disassembly at lipid droplet-associated regions of the ER. Released genomes would then be captured by core protein sequestered on the lipid droplet surface to enable initiation of virion morphogenesis [103,113]. Genome transfer may not be the only reason for the close proximity of replication complexes to lipid droplet-associated regions of the ER in virus-infected cells; an unexpected role for NS3 in virion morphogenesis at lipid droplets has been recently described [23]. Multiple mutations in NS3 can rescue a defect in an early-intermediate step in virus assembly that follows recruitment of NS5A to lipid droplet-associated ER regions but precedes the formation of intracellular virus particles [23]. Thus, it is possible that interaction of NS3 with a virus- or host-encoded protein at the lipid droplet surface may be involved in particle assembly and suggests that additional interactions involving the NS proteins, which serve to facilitate virion formation, may be identified in the future.

### 5.4. The link between assembly of HCV and VLDL

There are clear connections between HCV and VLDL assembly since inhibitors of MTP and decreasing expression of apoB by siRNA- and shRNA-targeting lowers virion production [116,117]. Lowering apoE expression also impairs release of infectious HCV [118,119] although the authors of these studies found that reducing apoB did not affect virion production and high levels of MTP inhibitors were necessary to block release of virus progeny. Further evidence of a role for apoE in virus secretion, which involved interaction with NS5A, has been reported recently [120]. It has been proposed that a selection process during assembly may direct nascent virus particles for degradation should they not undergo complete maturation that could involve addition of lipids [116]. However, any further details as to the mechanisms that operate during virion assembly are not known. With regard to lipid droplets, there is conclusive evidence that disabling the ability of core to target lipid droplets leads to impairment of infectious virus production [97]. Since lipid droplets do play a key role in VLDL assembly, it is reasonable to speculate that they actively participate in the production of virions. Indeed, the targeting of core to lipid droplets could represent an entry site for the virus to gain access to the VLDL assembly pathway.

## 6. The role of extracellular factors involved in lipid metabolism that participate in virus entry

Aside from intracellular processes that influence HCV RNA replication and virion assembly, extracellular components and factors on the plasma membrane, which are engaged in lipid transport, also play crucial roles in the entry of secreted, infectious virus into uninfected cells. This topic has been recently reviewed [122,123] and is briefly outlined in the following sections.

### 6.1. Receptors on the cell surface necessary for virus infection

The HCV entry pathway requires interaction between virion components, in particular the envelope glycoproteins E1 and E2, and several molecules on the cell surface. These include the low-density lipoprotein receptor (LDL-R) [124], glycosaminoglycans [125], CD81 [126], SR-BI [127], Claudin-1 (CLDN-1) [128] and occludin [129]. In the context of lipid transport, two surface receptors, LDL-R and SR-BI, are worthy of note.

LDL-R sequesters cholesterol-containing lipoproteins from the circulation for uptake of cholesterol into cells [130]. The receptor primarily binds LDL but it also has high affinity for VLDL particles containing multiple copies of apoE. Lipoprotein particles complexed to LDL-R are internalized by endocytosis via clathrin-coated pits and then transported to endosomes. The low pH environment in endosomes induces detachment of LDL-R from internalized lipoprotein particles, which are thereafter broken down by lysosomes to release cholesterol into the cell. HCV entry can be blocked by antibodies against LDL-R and a soluble peptide that encompasses the LDL-binding domain of the receptor [124,131]. Moreover, a recent report has demonstrated that reducing the abundance of LDL-R lowers infectivity of cell culture-derived virions, supporting the notion that interaction with the receptor contributes to productive infection [132]. The virion component that interacts with LDL-R is likely to be a cell-derived lipoprotein since antibodies against apoB and apoE disrupt infection [118,124,132,133].

SR-BI is a scavenger receptor that plays a key role in promoting the exchange of cholesterol between cells and lipoprotein particles [134]. The primary ligand for SR-BI is high-density lipoprotein (HDL) from which it selectively transfers cholesterol ester by a non-endocytic mechanism that, in contrast to cholesterol import by LDL-R, does not require degradation of HDL-associated apolipoproteins. SR-BI was initially identified as a potential receptor for HCV entry since it was capable of binding to the soluble portion of E2 [127] although direct interaction between SR-BI and the viral glycoproteins on infectious particles has not been demonstrated. Antibodies against SR-BI and reduced expression of the receptor inhibit HCV infection [135–138], providing confirmatory evidence for its contribution to virus entry.

### 6.2. Extracellular components that affect virus entry

As well as receptors expressed on the cell surface, extracellular components determine the efficiency of virus infection. For example, HDL, which is the ligand for SR-BI, enhances the process of entry through a mechanism that relies on the lipid transfer function of the receptor [135,138,139]. By contrast, oxidized LDL, another ligand for SR-BI, is a potent inhibitor of HCV infection [140] and VLDL also blocks the entry process [141]. Finally, lipoprotein lipase, the key enzyme involved in lipolysis of triglycerides in circulating VLDL, modulates cellular uptake of the virus [133].

## 7. Conclusions and future perspectives

**Figure 2.**
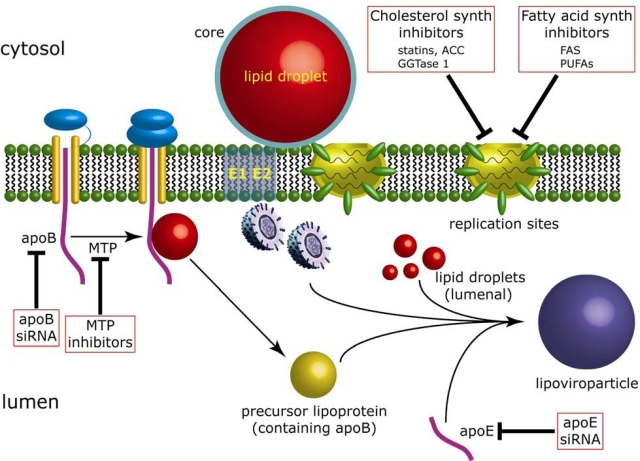
Inhibition of HCV RNA replication and virion assembly by blocking lipid biosynthesis and VLDL assembly. HCV RNA replication occurs at the ER membrane in specialized sites (the membranous web) that contain replication complexes. HCV core associates with cytosolic LDs and its interaction with NS5A at HCV RNA replication sites enables engagement of viral genomes with core to initiate virus assembly, possibly creating nucleocapids. Assembly is then thought to proceed through unknown processes in which the viral envelope glycoproteins (E1 and E2) are added to nucleocapsids and combine also with the VLDL assembly pathway to yield lipoviroparticles [121] that are released from the cell. Production of virus particles is thought to occur through interaction with the VLDL assembly pathway, which is a two-stage process. Firstly, there is initial lipidation of translocated apoB by MTP to create a pre-VLDL particle. Bulk triacylglycerol from cytosolic LDs is added to pre-VLDL particles through a process that is considered to produce lumenal LDs. Additional lipoprotein components are added also at this stage (including apoE) to generate mature VLDL. Steps in fatty acid and cholesterol biosynthesis and the VLDL assembly pathway that can be impaired thereby blocking HCV RNA replication and virion assembly are boxed. Abbreviations are as follows: ACC, acetyl-CoA carboxylase; FAS, fatty acid synthase; GGTase 1, geranylgeranyltransferase 1; MTP, microsomal transfer protein; PUFAs, polyunsaturated fatty acids.

In summary, there is conclusive evidence that the production of infectious virus from HCV-infected cells relies on several aspects of lipid metabolism (summarized in Figure 2). However, further studies are necessary to improve our understanding at the molecular level of the underlying mechanisms that engage metabolic pathways with both HCV RNA replication and virion assembly. In the case of RNA replication, the dependence on both fatty acid and cholesterol metabolism may indicate the need for a particular lipophilic environment to support genome synthesis. Creating such an environment may rely not only on lipid biosynthetic pathways but also factors involved in membrane organization such as class III phosphatidylinositol 4-kinase alpha (PI4K-IIIα), which plays a critical role in HCV RNA replication [142–144]. Moreover, additional studies into the biology of lipid droplets, and more in-depth analysis of the molecular requirements for the latter stages in the HCV life cycle, will help delineate the exact role for these organelles in the generation of HCV virions. Finally, the reliance of HCV upon both lipid biosynthesis and storage raises the distinct possibility that these topics could be important for development of new therapies for pharmacological inhibition of HCV morphogenesis.

## References

[1] The Global Burden of Hepatitis C Working Group (2004). Global burden of disease (GBD) for hepatitis C. J Clin Pharmacol.

[2] Alter MJ (2007). Epidemiology of hepatitis C virus infection. World J Gastroenterol.

[3] (1999). Global surveillance and control of hepatitis C. Report of a WHO Consultation organized in collaboration with the Viral Hepatitis Prevention Board, Antwerp, Belgium. J Viral Hepat.

[4] Di Bisceglie AM, Goodman ZD, Ishak KG, Hoofnagle JH, Melpolder JJ, Alter HJ (1991). Long-term clinical and histopathological follow-up of chronic posttransfusion hepatitis. Hepatology.

[5] Di Bisceglie AM (1998). Hepatitis C. Lancet.

[6] Di Bisceglie AM (1997). Hepatitis C and hepatocellular carcinoma. Hepatology.

[7] Robertson B, Myers G, Howard C, Brettin T, Bukh J, Gaschen B, Gojobori T, Maertens G, Mizokami M, Nainan O, Netesov S, Nishioka K, Shin i T, Simmonds P, Smith D, Stuyver L, Weiner A (1998). Classification, nomenclature, and database development for hepatitis C virus (HCV) and related viruses: proposals for standardization. International Committee on Virus Taxonomy. Arch Virol.

[8] Simmonds P, Bukh J, Combet C, Deleage G, Enomoto N, Feinstone S, Halfon P, Inchauspe G, Kuiken C, Maertens G, Mizokami M, Murphy DG, Okamoto H, Pawlotsky JM, Penin F, Sablon E, Shin IT, Stuyver LJ, Thiel HJ, Viazov S, Weiner AJ, Widell A (2005). Consensus proposals for a unified system of nomenclature of hepatitis C virus genotypes. Hepatology.

[9] Simmonds P, Holmes EC, Cha TA, Chan SW, McOmish F, Irvine B, Beall E, Yap PL, Kolberg J, Urdea MS (1993). Classification of hepatitis C virus into six major genotypes and a series of subtypes by phylogenetic analysis of the NS-5 region. J Gen Virol.

[10] Douglas MW, George J (2009). Molecular mechanisms of insulin resistance in chronic hepatitis C. World J Gastroenterol.

[11] Negro F, Clement S (2009). Impact of obesity, steatosis and insulin resistance on progression and response to therapy of hepatitis C. J Viral Hepat.

[12] Manns MP, Wedemeyer H, Cornberg M (2006). Treating viral hepatitis C: efficacy, side effects, and complications. Gut.

[13] Moradpour D, Penin F, Rice CM (2007). Replication of hepatitis C virus. Nat Rev Microbiol.

[14] Penin F, Dubuisson J, Rey FA, Moradpour D, Pawlotsky JM (2004). Structural biology of hepatitis C virus. Hepatology.

[15] Thomssen R, Bonk S, Propfe C, Heermann KH, Kochel HG, Uy A (1992). Association of hepatitis C virus in human sera with beta-lipoprotein. Med Microbiol Immunol (Berl).

[16] Thomssen R, Bonk S, Thiele A (1993). Density heterogeneities of hepatitis C virus in human sera due to the binding of beta-lipoproteins and immunoglobulins. Med Microbiol Immunol (Berl).

[17] Jones CT, Murray CL, Eastman DK, Tassello J, Rice CM (2007). Hepatitis C virus p7 and NS2 proteins are essential for production of infectious virus. J Virol.

[18] Steinmann E, Penin F, Kallis S, Patel AH, Bartenschlager R, Pietschmann T (2007). Hepatitis C virus p7 protein is crucial for assembly and release of infectious virions. PLoS Pathog.

[19] Appel N, Schaller T, Penin F, Bartenschlager R (2006). From structure to function: new insights into hepatitis C virus RNA replication. J Biol Chem.

[20] Bartenschlager R, Frese M, Pietschmann T (2004). Novel insights into hepatitis C virus replication and persistence. Adv Virus Res.

[21] Appel N, Zayas M, Miller S, Krijnse-Locker J, Schaller T, Friebe P, Kallis S, Engel U, Bartenschlager R (2008). Essential role of domain III of nonstructural protein 5A for hepatitis C virus infectious particle assembly. PLoS Pathog.

[22] Jones DM, Patel AH, Targett-Adams P, McLauchlan J (2009). The hepatitis C virus NS4B protein can trans-complement viral RNA replication and modulates production of infectious virus. J Virol.

[23] Ma Y, Yates J, Liang Y, Lemon SM, Yi M (2008). NS3 helicase domains involved in infectious intracellular hepatitis C virus particle assembly. J Virol.

[24] Tellinghuisen TL, Foss KL, Treadaway J (2008). Regulation of hepatitis C virion production via phosphorylation of the NS5A protein. PLoS Pathog.

[25] Yi M, Ma Y, Yates J, Lemon SM (2007). Compensatory mutations in E1, p7, NS2, and NS3 enhance yields of cell culture-infectious intergenotypic chimeric hepatitis C virus. J Virol.

[26] Gosert R, Egger D, Lohmann V, Bartenschlager R, Blum HE, Bienz K, Moradpour D (2003). Identification of the hepatitis C virus RNA replication complex in Huh-7 cells harboring subgenomic replicons. J Virol.

[27] Rust RC, Landmann L, Gosert R, Tang BL, Hong W, Hauri HP, Egger D, Bienz K (2001). Cellular COPII proteins are involved in production of the vesicles that form the poliovirus replication complex. J Virol.

[28] Suhy DA, Giddings TH, Kirkegaard K (2000). Remodeling the endoplasmic reticulum by poliovirus infection and by individual viral proteins: an autophagy-like origin for virus-induced vesicles. J Virol.

[29] Smith S, Witkowski A, Joshi AK (2003). Structural and functional organization of the animal fatty acid synthase. Prog Lip Res.

[30] Maier T, Jenni S, Ban N (2006). Architecture of mammalian fatty acid synthase at 4.5 A resolution. Science.

[31] Guillou H, Zadravec D, Martin PG, Jacobsson A (2010). The key roles of elongases and desaturases in mammalian fatty acid metabolism: Insights from transgenic mice. Prog Lip Res.

[32] Yen CL, Stone SJ, Koliwad S, Harris C, Farese RV (2008). Thematic review series: glycerolipids. DGAT enzymes and triacylglycerol biosynthesis. J Lip Res.

[33] Shi Y, Cheng D (2009). Beyond triglyceride synthesis: the dynamic functional roles of MGAT and DGAT enzymes in energy metabolism. Am J Physiol.

[34] McTaggart SJ (2006). Isoprenylated proteins. Cell Mol Life Sci.

[35] Chang TY, Li BL, Chang CC, Urano Y (2009). Acyl-coenzyme A:cholesterol acyltransferases. Am J Physiol.

[36] Tauchi-Sato K, Ozeki S, Houjou T, Taguchi R, Fujimoto T (2002). The surface of lipid droplets is a phospholipid monolayer with a unique fatty Acid composition. J Biol Chem.

[37] Martin S, Parton RG (2006). Lipid droplets: a unified view of a dynamic organelle. Nat Rev Mol Cell Biol.

[38] Bickel PE, Tansey JT, Welte MA (2009). PAT proteins, an ancient family of lipid droplet proteins that regulate cellular lipid stores. Biochim Biophys Acta.

[39] Olofsson SO, Bostrom P, Andersson L, Rutberg M, Perman J, Boren J (2009). Lipid droplets as dynamic organelles connecting storage and efflux of lipids. Biochim Biophys Acta.

[40] Mahley RW, Innerarity TL, Rall SC, Weisgraber KH (1984). Plasma lipoproteins: apolipoprotein structure and function. J Lip Res.

[41] Kane JP (1983). Apolipoprotein B: structural and metabolic heterogeneity. Annu Rev Physiol.

[42] Elovson J, Chatterton JE, Bell GT, Schumaker VN, Reuben MA, Puppione DL, Reeve JR, Young NL (1988). Plasma very low density lipoproteins contain a single molecule of apolipoprotein B. J Lip Res.

[43] Wetterau JR, Combs KA, Spinner SN, Joiner BJ (1990). Protein disulfide isomerase is a component of the microsomal triglyceride transfer protein complex. J Biol Chem.

[44] Hussain MM, Shi J, Dreizen P (2003). Microsomal triglyceride transfer protein and its role in apoB-lipoprotein assembly. J Lip Res.

[45] Kulinski A, Rustaeus S, Vance JE (2002). Microsomal triacylglycerol transfer protein is required for lumenal accretion of triacylglycerol not associated with ApoB, as well as for ApoB lipidation. J Biol Chem.

[46] Pan M, Liang J-S, Fisher EA, Ginsberg HN (2002). The late addition of core lipids to nascent apolipoprotein B100, resulting in the assembly and secretion of triglyceride-rich lipoproteins, is independent of both microsomal triglyceride transfer protein activity and new triglyceride synthesis. J Biol Chem.

[47] Gibbons GF, Wiggins D, Brown AM, Hebbachi AM (2004). Synthesis and function of hepatic very-low-density lipoprotein. Biochem Soc Trans.

[48] Fujimoto Y, Itabe H, Sakai J, Makita M, Noda J, Mori M, Higashi Y, Kojima S, Takano T (2004). Identification of major proteins in the lipid droplet-enriched fraction isolated from the human hepatocyte cell line HuH7. Biochim Biophys Acta.

[49] Magnusson B, Asp L, Bostrom P, Ruiz M, Stillemark-Billton P, Linden D, Boren J, Olofsson SO (2006). Adipocyte differentiation-related protein promotes fatty acid storage in cytosolic triglycerides and inhibits secretion of very low-density lipoproteins. Arterioscler Thromb Vasc Biol.

[50] Lehner R, Vance DE (1999). Cloning and expression of a cDNA encoding a hepatic microsomal lipase that mobilizes stored triacylglycerol. Biochem J.

[51] Wei E, Alam M, Sun F, Agellon LB, Vance DE, Lehner R (2007). Apolipoprotein B and triacylglycerol secretion in human triacylglycerol hydrolase transgenic mice. J Lip Res.

[52] Yamazaki T, Sasaki E, Kakinuma C, Yano T, Miura S, Ezaki O (2005). Increased very low density lipoprotein secretion and gonadal fat mass in mice overexpressing liver DGAT1. J Biol Chem.

[53] Buhman KF, Accad M, Farese RV (2000). Mammalian acyl-CoA:cholesterol acyltransferases. Biochim Biophys Acta.

[54] Buhman KK, Accad M, Novak S, Choi RS, Wong JS, Hamilton RL, Turley S, Farese RV (2000). Resistance to diet-induced hypercholesterolemia and gallstone formation in ACAT2-deficient mice. Nat Med.

[55] Liang JJ, Oelkers P, Guo C, Chu PC, Dixon JL, Ginsberg HN, Sturley SL (2004). Overexpression of human diacylglycerol acyltransferase 1, acyl-coa:cholesterol acyltransferase 1, or acyl-CoA:cholesterol acyltransferase 2 stimulates secretion of apolipoprotein B-containing lipoproteins in McA-RH7777 cells. J Biol Chem.

[56] Millar JS, Stone SJ, Tietge UJ, Tow B, Billheimer JT, Wong JS, Hamilton RL, Farese RV, Rader DJ (2006). Short-term overexpression of DGAT1 or DGAT2 increases hepatic triglyceride but not VLDL triglyceride or apoB production. J Lip Res.

[57] Hussy P, Langen H, Mous J, Jacobsen H (1996). Hepatitis C virus core protein: carboxy-terminal boundaries of two processed species suggest cleavage by a signal peptide peptidase. Virology.

[58] McLauchlan J, Lemberg MK, Hope G, Martoglio B (2002). Intramembrane proteolysis promotes trafficking of hepatitis C virus core protein to lipid droplets. Embo J.

[59] Santolini E, Migliaccio G, La Monica N (1994). Biosynthesis and biochemical properties of the hepatitis C virus core protein. J Virol.

[60] Ogino T, Fukuda H, Imajoh-Ohmi S, Kohara M, Nomoto A (2004). Membrane binding properties and terminal residues of the mature hepatitis C virus capsid protein in insect cells. J. Virol..

[61] McLauchlan J (2009). Lipid droplets and hepatitis C virus infection. Biochim Biophys Acta.

[62] McLauchlan J (2000). Properties of the hepatitis C virus core protein: a structural protein that modulates cellular processes. J Viral Hepat.

[63] Boulant S, Montserret R, Hope RG, Ratinier M, Targett-Adams P, Lavergne JP, Penin F, McLauchlan J (2006). Structural determinants that target the hepatitis C virus core protein to lipid droplets. J Biol Chem.

[64] Boulant S, Vanbelle C, Ebel C, Penin F, Lavergne JP (2005). Hepatitis C virus core protein is a dimeric alpha-helical protein exhibiting membrane protein features. J Virol.

[65] Tellinghuisen TL, Marcotrigiano J, Gorbalenya AE, Rice CM (2004). The NS5A protein of hepatitis C virus is a zinc metalloprotein. J Biol Chem.

[66] Appel N, Pietschmann T, Bartenschlager R (2005). Mutational analysis of hepatitis C virus nonstructural protein 5A: potential role of differential phosphorylation in RNA replication and identification of a genetically flexible domain. J Virol.

[67] Moradpour D, Evans MJ, Gosert R, Yuan Z, Blum HE, Goff SP, Lindenbach BD, Rice CM (2004). Insertion of green fluorescent protein into nonstructural protein 5A allows direct visualization of functional hepatitis C virus replication complexes. J Virol.

[68] Tellinghuisen TL, Foss KL, Treadaway JC, Rice CM (2008). Identification of residues required for RNA replication in domains II and III of the hepatitis C virus NS5A protein. J Virol.

[69] Tellinghuisen TL, Marcotrigiano J, Rice CM (2005). Structure of the zinc-binding domain of an essential component of the hepatitis C virus replicase. Nature.

[70] Brass V, Bieck E, Montserret R, Wolk B, Hellings JA, Blum HE, Penin F, Moradpour D (2002). An amino-terminal amphipathic alpha-helix mediates membrane association of the hepatitis C virus nonstructural protein 5A. J Biol Chem.

[71] Penin F, Brass V, Appel N, Ramboarina S, Montserret R, Ficheux D, Blum HE, Bartenschlager R, Moradpour D (2004). Structure and function of the membrane anchor domain of hepatitis C virus nonstructural protein 5A. J Biol Chem.

[72] Shi ST, Polyak SJ, Tu H, Taylor DR, Gretch DR, Lai MM (2002). Hepatitis C virus NS5A colocalizes with the core protein on lipid droplets and interacts with apolipoproteins. Virology.

[73] Miyanari Y, Atsuzawa K, Usuda N, Watashi K, Hishiki T, Zayas M, Bartenschlager R, Wakita T, Hijikata M, Shimotohno K (2007). The lipid droplet is an important organelle for hepatitis C virus production. Nat Cell Biol.

[74] Bigger CB, Brasky KM, Lanford RE (2001). DNA microarray analysis of chimpanzee liver during acute resolving hepatitis C virus infection. J Virol.

[75] Bigger CB, Guerra B, Brasky KM, Hubbard G, Beard MR, Luxon BA, Lemon SM, Lanford RE (2004). Intrahepatic gene expression during chronic hepatitis C virus infection in chimpanzees. J Virol.

[76] Su AI, Pezacki JP, Wodicka L, Brideau AD, Supekova L, Thimme R, Wieland S, Bukh J, Purcell RH, Schultz PG, Chisari FV (2002). Genomic analysis of the host response to hepatitis C virus infection. Proc Natl Acad Sci USA.

[77] Kapadia SB, Chisari FV (2005). Hepatitis C virus RNA replication is regulated by host geranylgeranylation and fatty acids. Proc Natl Acad Sci USA.

[78] Ye J, Wang C, Sumpter R, Brown MS, Goldstein JL, Gale M (2003). Disruption of hepatitis C virus RNA replication through inhibition of host protein geranylgeranylation. Proc Natl Acad Sci USA.

[79] Delang L, Paeshuyse J, Vliegen I, Leyssen P, Obeid S, Durantel D, Zoulim F, Op de Beeck A, Neyts J (2009). Statins potentiate the *in vitro* anti-hepatitis C virus activity of selective hepatitis C virus inhibitors and delay or prevent resistance development. Hepatology.

[80] Ikeda M, Abe K, Yamada M, Dansako H, Naka K, Kato N (2006). Different anti-HCV profiles of statins and their potential for combination therapy with interferon. Hepatology.

[81] Bader T, Fazili J, Madhoun M, Aston C, Hughes D, Rizvi S, Seres K, Hasan M (2008). Fluvastatin inhibits hepatitis C replication in humans. Am J Gastroenterol.

[82] Madhoun MF, Bader T (2010). Statins improve ALT values in chronic hepatitis C patients with abnormal values. Dig Dis Sci.

[83] Milazzo L, Caramma I, Mazzali C, Cesari M, Olivetti M, Galli M, Antinori S (2010). Fluvastatin as an adjuvant to pegylated interferon and ribavirin in HIV/hepatitis C virus genotype 1 co-infected patients: an open-label randomized controlled study. J Antimicrob Chemother.

[84] Forde KA, Law C, O'Flynn R, Kaplan DE (2009). Do statins reduce hepatitis C RNA titers during routine clinical use. World J Gastroenterol.

[85] O'Leary JG, Chan JL, McMahon CM, Chung RT (2007). Atorvastatin does not exhibit antiviral activity against HCV at conventional doses: a pilot clinical trial. Hepatology.

[86] Wang C, Gale M, Keller BC, Huang H, Brown MS, Goldstein JL, Ye J (2005). Identification of FBL2 as a geranylgeranylated cellular protein required for hepatitis C virus RNA replication. Mol Cell.

[87] Leu GZ, Lin TY, Hsu JT (2004). Anti-HCV activities of selective polyunsaturated fatty acids. Biochem Biophys Res Commun.

[88] Huang H, Chen Y, Ye J (2007). Inhibition of hepatitis C virus replication by peroxidation of arachidonate and restoration by vitamin E. Proc Natl Acad Sci USA.

[89] Westaway EG, Mackenzie JM, Kenney MT, Jones MK, Khromykh AA (1997). Ultrastructure of Kunjin virus-infected cells: colocalization of NS1 and NS3 with double-stranded RNA, and of NS2B with NS3, in virus-induced membrane structures. J Virol.

[90] Roingeard P, Hourioux C, Blanchard E, Prensier G (2008). Hepatitis C virus budding at lipid droplet-associated ER membrane visualized by 3D electron microscopy. Histochem Cell Biol.

[91] Zhong J, Gastaminza P, Cheng G, Kapadia S, Kato T, Burton DR, Wieland SF, Uprichard SL, Wakita T, Chisari FV (2005). Robust hepatitis C virus infection *in vitro*. Proc Natl Acad Sci USA.

[92] Wakita T, Pietschmann T, Kato T, Date T, Miyamoto M, Zhao Z, Murthy K, Habermann A, Krausslich HG, Mizokami M, Bartenschlager R, Liang TJ (2005). Production of infectious hepatitis C virus in tissue culture from a cloned viral genome. Nat Med.

[93] Lindenbach BD, Evans MJ, Syder AJ, Wolk B, Tellinghuisen TL, Liu CC, Maruyama T, Hynes RO, Burton DR, McKeating JA, Rice CM (2005). Complete replication of hepatitis C virus in cell culture. Science.

[94] Targett-Adams P, Boulant S, McLauchlan J (2008). Visualization of double-stranded RNA in cells supporting hepatitis C virus RNA replication. J Virol.

[95] Ma Y, Yates J, Liang Y, Lemon SM, Yi M (2008). NS3 helicase domains involved in infectious intracellular hepatitis C virus particle assembly. J Virol.

[96] Rouille Y, Helle F, Delgrange D, Roingeard P, Voisset C, Blanchard E, Belouzard S, McKeating J, Patel AH, Maertens G, Wakita T, Wychowski C, Dubuisson J (2006). Subcellular localization of hepatitis C virus structural proteins in a cell culture system that efficiently replicates the virus. J Virol.

[97] Targett-Adams P, Hope G, Boulant S, McLauchlan J (2008). Maturation of hepatitis C virus core protein by signal peptide peptidase is required for virus production. J Biol Chem.

[98] Boulant S, Douglas MW, Moody L, Budkowska A, Targett-Adams P, McLauchlan J (2008). Hepatitis C virus core protein induces lipid droplet redistribution in a microtubule- and dynein-dependent manner. Traffic.

[99] Shavinskaya A, Boulant S, Penin F, McLauchlan J, Bartenschlager R (2007). The lipid droplet binding domain of hepatitis C virus core protein is a major determinant for efficient virus assembly. J Biol Chem.

[100] Blight KJ, Kolykhalov AA, Rice CM (2000). Efficient initiation of HCV RNA replication in cell culture. Science.

[101] Lohmann V, Korner F, Koch J, Herian U, Theilmann L, Bartenschlager R (1999). Replication of subgenomic hepatitis C virus RNAs in a hepatoma cell line. Science.

[102] Moradpour D, Brass V, Penin F (2005). Function follows form: the structure of the N-terminal domain of HCV NS5A. Hepatology.

[103] Masaki T, Suzuki R, Murakami K, Aizaki H, Ishii K, Murayama A, Date T, Matsuura Y, Miyamura T, Wakita T, Suzuki T (2008). Interaction of hepatitis C virus nonstructural protein 5A with core protein is critical for the production of infectious virus particles. J Virol.

[104] Kaul A, Stauffer S, Berger C, Pertel T, Schmitt J, Kallis S, Zayas M, Lohmann V, Luban J, Bartenschlager R (2009). Essential role of cyclophilin A for hepatitis C virus replication and virus production and possible link to polyprotein cleavage kinetics. PLoS Pathog.

[105] Watashi K, Ishii N, Hijikata M, Inoue D, Murata T, Miyanari Y, Shimotohno K (2005). Cyclophilin B is a functional regulator of hepatitis C virus RNA polymerase. Mol Cell.

[106] Yang F, Robotham JM, Nelson HB, Irsigler A, Kenworthy R, Tang H (2008). Cyclophilin A is an essential cofactor for hepatitis C virus infection and the principal mediator of cyclosporine resistance i*n vitro*. J Virol.

[107] Huang L, Hwang J, Sharma SD, Hargittai MR, Chen Y, Arnold JJ, Raney KD, Cameron CE (2005). Hepatitis C virus nonstructural protein 5A (NS5A) is an RNA-binding protein. J Biol Chem.

[108] Tong X, Malcolm BA (2006). Trans-complementation of HCV replication by non-structural protein 5A. Virus Res.

[109] Appel N, Herian U, Bartenschlager R (2005). Efficient rescue of hepatitis C virus RNA replication by trans-complementation with nonstructural protein 5A. J Virol.

[110] Tanji Y, Kaneko T, Satoh S, Shimotohno K (1995). Phosphorylation of hepatitis C virus-encoded nonstructural protein NS5A. J Virol.

[111] Reed KE, Xu J, Rice CM (1997). Phosphorylation of the hepatitis C virus NS5A protein *in vitro* and *in vivo*: properties of the NS5A-associated kinase. J Virol.

[112] Reed KE, Rice CM (1999). Identification of the major phosphorylation site of the hepatitis C virus H strain NS5A protein as serine 2321. J Biol Chem.

[113] Evans MJ, Rice CM, Goff SP (2004). Phosphorylation of hepatitis C virus nonstructural protein 5A modulates its protein interactions and viral RNA replication. Proc Natl Acad Sci USA.

[114] Neddermann P, Quintavalle M, Di Pietro C, Clementi A, Cerretani M, Altamura S, Bartholomew L, De Francesco R (2004). Reduction of hepatitis C virus NS5A hyperphosphorylation by selective inhibition of cellular kinases activates viral RNA replication in cell culture. J Virol.

[115] Lohmann V, Hoffmann S, Herian U, Penin F, Bartenschlager R (2003). Viral and cellular determinants of hepatitis C virus RNA replication in cell culture. J Virol.

[116] Gastaminza P, Cheng G, Wieland S, Zhong J, Liao W, Chisari FV (2008). Cellular determinants of hepatitis C virus assembly, maturation, degradation, and secretion. J Virol.

[117] Huang H, Sun F, Owen DM, Li W, Chen Y, Gale M, Ye J (2007). Hepatitis C virus production by human hepatocytes dependent on assembly and secretion of very low-density lipoproteins. Proc Natl Acad Sci USA.

[118] Chang KS, Jiang J, Cai Z, Luo G (2007). Human apolipoprotein E is required for infectivity and production of hepatitis C virus in cell culture. J Virol.

[119] Jiang J, Luo G (2009). Apolipoprotein E but not B is required for the formation of infectious hepatitis C virus particles. J Virol.

[120] Benga WJ, Krieger SE, Dimitrova M, Zeisel MB, Parnot M, Lupberger J, Hildt E, Luo G, McLauchlan J, Baumert TF, Schuster C (2010). Apolipoprotein E interacts with hepatitis C virus nonstructural protein 5A and determines assembly of infectious particles. Hepatology.

[121] Andre P, Komurian-Pradel F, Deforges S, Perret M, Berland JL, Sodoyer M, Pol S, Brechot C, Paranhos-Baccala G, Lotteau V (2002). Characterization of low- and very-low-density hepatitis C virus RNA-containing particles. J Virol.

[122] Bartosch B, Dubuisson J (2010). Recent advances in hepatitis C virus cell entry. Viruses.

[123] Burlone ME, Budkowska A (2009). Hepatitis C virus cell entry: role of lipoproteins and cellular receptors. J Gen Virol.

[124] Agnello V, Abel G, Elfahal M, Knight GB, Zhang QX (1999). Hepatitis C virus and other flaviviridae viruses enter cells via low density lipoprotein receptor. Proc Natl Acad Sci USA.

[125] Germi R, Crance JM, Garin D, Guimet J, Lortat-Jacob H, Ruigrok RW, Zarski JP, Drouet E (2002). Cellular glycosaminoglycans and low density lipoprotein receptor are involved in hepatitis C virus adsorption. J Med Virol.

[126] Pileri P, Uematsu Y, Campagnoli S, Galli G, Falugi F, Petracca R, Weiner AJ, Houghton M, Rosa D, Grandi G, Abrignani S (1998). Binding of hepatitis C virus to CD81. Science.

[127] Scarselli E, Ansuini H, Cerino R, Roccasecca RM, Acali S, Filocamo G, Traboni C, Nicosia A, Cortese R, Vitelli A (2002). The human scavenger receptor class B type I is a novel candidate receptor for the hepatitis C virus. Embo J.

[128] Evans MJ, von Hahn T, Tscherne DM, Syder AJ, Panis M, Wolk B, Hatziioannou T, McKeating JA, Bieniasz PD, Rice CM (2007). Claudin-1 is a hepatitis C virus co-receptor required for a late step in entry. Nature.

[129] Ploss A, Evans MJ, Gaysinskaya VA, Panis M, You H, de Jong YP, Rice CM (2009). Human occludin is a hepatitis C virus entry factor required for infection of mouse cells. Nature.

[130] Beglova N, Blacklow SC (2005). The LDL receptor: how acid pulls the trigger. Trends Biochem Sci.

[131] Molina S, Castet V, Fournier-Wirth C, Pichard-Garcia L, Avner R, Harats D, Roitelman J, Barbaras R, Graber P, Ghersa P, Smolarsky M, Funaro A, Malavasi F, Larrey D, Coste J, Fabre JM, Sa-Cunha A, Maurel P (2007). The low-density lipoprotein receptor plays a role in the infection of primary human hepatocytes by hepatitis C virus. J Hepatol.

[132] Owen DM, Huang H, Ye J, Gale M (2009). Apolipoprotein E on hepatitis C virion facilitates infection through interaction with low-density lipoprotein receptor. Virology.

[133] Andreo U, Maillard P, Kalinina O, Walic M, Meurs E, Martinot M, Marcellin P, Budkowska A (2007). Lipoprotein lipase mediates hepatitis C virus (HCV) cell entry and inhibits HCV infection. Cell Microbiol.

[134] Connelly MA, Williams DL (2004). Scavenger receptor BI: a scavenger receptor with a mission to transport high density lipoprotein lipids. Curr Opin Lipidol.

[135] Catanese MT, Graziani R, von Hahn T, Moreau M, Huby T, Paonessa G, Santini C, Luzzago A, Rice CM, Cortese R, Vitelli A, Nicosia A (2007). High-avidity monoclonal antibodies against the human scavenger class B type I receptor efficiently block hepatitis C virus infection in the presence of high-density lipoprotein. J Virol.

[136] Grove J, Huby T, Stamataki Z, Vanwolleghem T, Meuleman P, Farquhar M, Schwarz A, Moreau M, Owen JS, Leroux-Roels G, Balfe P, McKeating JA (2007). Scavenger receptor BI and BII expression levels modulate hepatitis C virus infectivity. J Virol.

[137] Kapadia SB, Barth H, Baumert T, McKeating JA, Chisari FV (2007). Initiation of hepatitis C virus infection is dependent on cholesterol and cooperativity between CD81 and scavenger receptor B type I. J Virol.

[138] Zeisel MB, Koutsoudakis G, Schnober EK, Haberstroh A, Blum HE, Cosset FL, Wakita T, Jaeck D, Doffoel M, Royer C, Soulier E, Schvoerer E, Schuster C, Stoll-Keller F, Bartenschlager R, Pietschmann T, Barth H, Baumert TF (2007). Scavenger receptor class B type I is a key host factor for hepatitis C virus infection required for an entry step closely linked to CD81. Hepatology.

[139] Voisset C, Callens N, Blanchard E, Op De Beeck A, Dubuisson J, Vu-Dac N (2005). High density lipoproteins facilitate hepatitis C virus entry through the scavenger receptor class B type I. J Biol Chem.

[140] von Hahn T, Lindenbach BD, Boullier A, Quehenberger O, Paulson M, Rice CM, McKeating JA (2006). Oxidized low-density lipoprotein inhibits hepatitis C virus cell entry in human hepatoma cells. Hepatology.

[141] Maillard P, Huby T, Andreo U, Moreau M, Chapman J, Budkowska A (2006). The interaction of natural hepatitis C virus with human scavenger receptor SR-BI/Cla1 is mediated by ApoB-containing lipoproteins. FASEB J.

[142] Berger KL, Cooper JD, Heaton NS, Yoon R, Oakland TE, Jordan TX, Mateu G, Grakoui A, Randall G (2009). Roles for endocytic trafficking and phosphatidylinositol 4-kinase III alpha in hepatitis C virus replication. Proc Natl Acad Sci USA.

[143] Borawski J, Troke P, Puyang X, Gibaja V, Zhao S, Mickanin C, Leighton-Davies J, Wilson CJ, Myer V, Cornellataracido I, Baryza J, Tallarico J, Joberty G, Bantscheff M, Schirle M, Bouwmeester T, Mathy JE, Lin K, Compton T, Labow M, Wiedmann B, Gaither LA (2009). Class III phosphatidylinositol 4-kinase alpha and beta are novel host factor regulators of hepatitis C virus replication. J Virol.

[144] Vaillancourt FH, Pilote L, Cartier M, Lippens J, Liuzzi M, Bethell RC, Cordingley MG, Kukolj G (2009). Identification of a lipid kinase as a host factor involved in hepatitis C virus RNA replication. Virology.

